# Report of the Seventh Post-Kala-Azar Dermal Leishmaniasis Consortium meeting, Kolkata, India, 28–29 November 2024

**DOI:** 10.1186/s13071-025-07175-2

**Published:** 2026-03-17

**Authors:** Mitali Chatterjee, Ritika Sengupta, Kristien Cloots, Eduard E. Zijlstra

**Affiliations:** 1 Department of Pharmacology, Institute of Postgraduate Medical Education and Research (IPGMER), Kolkata, India; 2 Department of Public Health, Institute of Tropical Medicine, Antwerp, Belgium; 3 Rotterdam Center for Tropical Medicine, Bovenstraat 21, 3077 BB Rotterdam, The Netherlands

**Keywords:** Post-kala-azar dermal leishmaniasis, Epidemiology, Diagnosis, Immunology, Treatment, Visceral leishmaniasis

## Abstract

The Post-Kala-Azar Dermal Leishmaniasis (PKDL) Consortium convened its seventh meeting from 28 to 29 November 2024 in Kolkata, India, to advance research and public health strategies related to PKDL and visceral leishmaniasis. This report summarizes the key discussions and outcomes of the meeting and proposes a way forward for addressing the last-mile challenge of eliminating leishmaniasis as a public health problem in South Asia and subsequently East Africa.

**Supplementary Information:**

The online version contains supplementary material available at 10.1186/s13071-025-07175-2.

## Background

The Post-Kala-Azar Dermal Leishmaniasis (PKDL) Consortium was founded in 2012 by the Drugs for Neglected Diseases *initiative* (DND*i*), based in Geneva, Switzerland, and Program for Appropriate Technology in Health (PATH), based in New Delhi, India. The Consortium aims to promote research on PKDL in all clinical and public health aspects to contribute to its improved management and control. The seventh meeting of the Consortium was held from 28 to 29 November 2024 in Kolkata, India. Two previous meeting reports have been published [1, 2], and the present report summarizes the proceedings of the most recent meeting. The agenda of the meeting and list of participants are provided in Additional file 1: Text S1; Additional file 2: Text S2.

The Kolkata meeting celebrated the scientific contributions of Sir Upendranath Brahmachari (1873–1946), Professor of Tropical Medicine, Carmichael Medical School, Calcutta, who was the first to confirm the diagnosis of PKDL in a landmark publication titled “A new form of cutaneous leishmaniasis-dermal leishmanoid” that was published in the* Indian Medical Gazette* in 1922 [3]. In addition, Sir UN Brahmachari contributed to the development of pentavalent antimony, which, over the years, has saved thousands of patients affected by visceral leishmaniasis (VL) [4–6]. The meeting was divided into six sessions encompassing various aspects of PKDL that included its epidemiology and potential control, immunological features, co-infections with PKDL and its diagnosis and treatment, including vaccines and chemotherapy.

## Session 1A: epidemiology and control of PKDL (presenters: Suman Rijal, Saurabh Jain, Aya Yajima, Dinesh Mondal, Abate Beshah, Eduard Zijlstra)

**Fig. 1:**
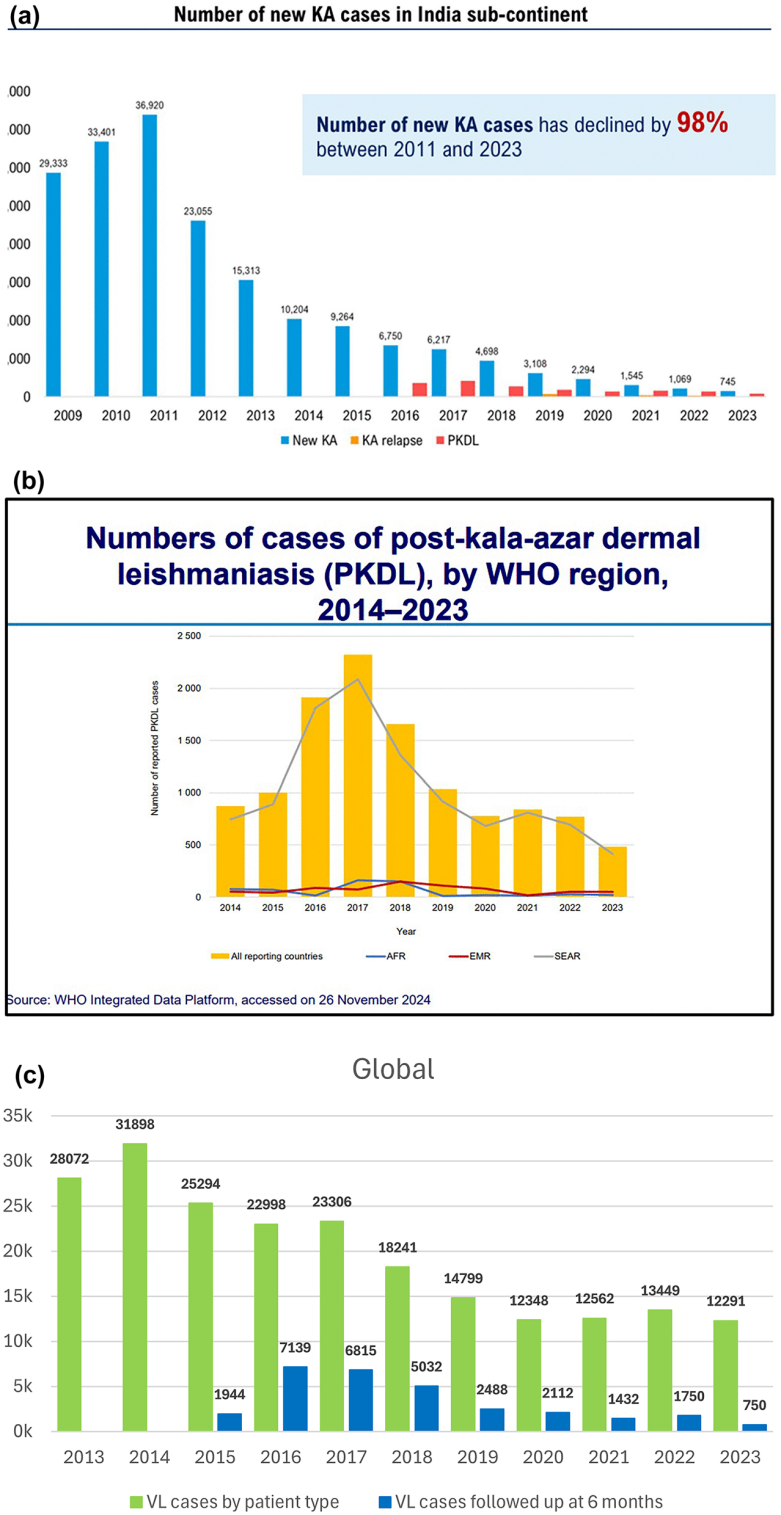
**a** Bar graphs showing the number of kala-azar (KA, new and relapsing) and post kala-azar dermal leishmaniasis (PKDL) cases between 2009 and 2024 in the Indian subcontinent. Yajima A, Status of VL control in Indian subcontinent ISC –focus on PKDL, 7th PKDL consortium, Kolkata, India, 28th-29th November 2024. **b** Bar graphs showing the number of post kala-azar dermal leishmaniasis (PKDL) cases across different WHO regions between 2014 and 2023. AFR: African Region, EMR: Eastern Mediterranean Region, SEAR: Southeast Asia Region. Source: https://www.who.int/publications/i/item/who-wer-9945-653-669. **c** Bar graphs indicating the number of VL cases (green) versus the number of VL cases followed up at the 6-month time point (blue) between 2013 and 2023. Source: WHO Integrated data platform, accessed on 26 November, 2024. Jain Saurabh, Global burden of PKDL, 7th PKDL consortium, Kolkata, India, 28th-29th November 2024. AFR, Africa; EMR, WHO Eastern Mediterranean Region; KA, kala-azar; PKDL, post-kala-azar dermal leishmaniasis; SEAR, WHO Southeast Asia Region; VL, visceral leishmaniasis

The strategic shift from millennium development goals to sustainable development goals (SDGs) prompted a focus on communicable diseases such as neglected tropical diseases (NTDs), including VL and skin NTDs (such as PKDL), in the Southeast Asia Region (SEAR), for which a holistic approach is essential. This has translated into tremendous progress over the past 20 years, as the number of VL cases in the SEAR has declined by 98% since 2011 (Fig. 1, top). Bangladesh was the first country in the world to reach the set target for VL elimination in 2023; India has reached the elimination threshold in all its endemic blocks and in Nepal, only two districts remain above the elimination target. However, in the long run, several challenges remain to be overcome, including a lack of sustainable funding, limited basic infrastructure, limited health system capacity, geopolitical instability, a lack of appropriate tools and innovation and climate change. Strategic approaches to address these challenges include country ownership, people-centered multisectoral approaches and community engagement, with the aim to address risk factors and foster regional partnerships for innovation and research (Fig. 1a; Additional file 3: Text S3).

Since 2017, particularly in the SEAR, the global number of PKDL cases has shown an overall downward trend (Fig. 1b), with most cases occurring in individuals in the age group ≥ 15 years and cases showing a male to female ratio of 1.4–1.8:1. Since 2016, the number of PKDL cases has fluctuated; for example, in India, the trend has consistently decreased, whereas in Bangladesh and Nepal, it has increased after a previous all-time low in 2021. However, for Nepal, the numbers are too low to draw meaningful conclusions. The notably higher proportion of pediatric cases observed in Nepal compared to India and Bangladesh represents a significant concern. In all three countries, a higher proportion of kala-azar cases were reported in males as compared to females, and this disparity can be attributed to gender differences in biological susceptibility, behavioral or occupational exposures and healthcare-seeking behavior. It is important to understand the number of expected PKDL cases in order to monitor progress toward the NTD 2030 target of detecting and treating all PKDL cases in the SEAR [7]. Recent data from Bangladesh revealed that 10% of VL patients who are cured develop PKDL, with a median lag period of 2.3 years, and that the PKDL burden is 1.3 per 100,000 people from villages endemic for VL [8]. As 10% of PKDL patients reported no history of VL, collaboration/integration with other dermatological services, such as leprosy hospitals, was required to reach these patients.

Over the last 10 years, the number of reported VL cases in the African region has decreased by 61% (11,119 in 2014 vs 4323 in 2023) (Fig. 1c). At present, 33% of the global burden of VL is reported from Africa, especially East Africa (EA). In 2024, a strategic framework was launched for the elimination of VL as a public health problem by 2030, with the challenges being inadequate resources, the impact of climate change and limited access to healthcare. The number of reported PKDL cases is low, with 27, eight, 48 and four cases reported from Ethiopia, Kenya, South Sudan and Uganda, respectively, between 2019 and 2023. It is likely that PKDL is underreported and underdiagnosed in these countries, as the follow-up of VL patients is poor, and many patients with PKDL tend to self-cure. The current interventions include an integrated skin NTD approach (which includes PKDL) in areas endemic for VL, awareness and educational programs targeting communities and research for improved diagnosis and treatment. In a recent hospital-based study in Sudan, 26% of VL patients who were cured developed PKDL, with a median time of 2 months after VL. This finding contrasts with historical data from home-based treatment, which reported that PKDL rates are 50–60%, and are possibly attributable to the administration of adulterated sodium stibogluconate (SSG) [9].

PKDL patients in SEAR often face significant social stigma and discrimination due to visible skin lesions, and the resemblance of these lesions to those of leprosy exacerbates the likelihood of ostracization. Addressing this stigma is an important aspect of providing patients with comprehensive care and support [8]. This sharply contrasts with the situation in Sudan, where the occurrence of PKDL (usually concomitantly with or within 1 year after VL) is considered a good sign (“the disease [kala-azar] has come-out”), as it implies that the child will live and not die from VL. Universal access to diagnostic tools and an active case search for VL have increased detection rates in high-risk regions such as Uganda and Sudan. Moreover, service decentralization has led to a reduction in fatality rates and improved treatment accessibility. Current challenges for VL and PKDL worldwide include the decreasing the percentage of VL patients who report for follow-up after 6 months from 30% to < 15% (Fig. 1, bottom). It was recommended that to achieve and sustain kala-azar elimination as a public health concern, the focus must be on optimizing, integrating and innovating the delivery of three core intervention, namely: (i) kala-azar and PKDL surveillance; (ii) comprehensive case management; and (iii) integrated vector management (IVM). This involves enhancing active case detection with advanced diagnostics, ensuring effective treatment regimens and combining indoor residual spraying with environmental and novel vector control measures. Sustained elimination requires robust, real-time surveillance systems, health system integration and community engagement to maintain interventions beyond validation. Innovative research into stable therapeutics and regional partnerships was recommended as critical for addressing challenges like asymptomatic reservoirs and insecticide resistance, with a view to ensuring long-term success.

## Session 1B: epidemiology and control of PKDL (presenters: Eduard E. Zijlstra, Dia El-Naiem, Om Prakash Singh)

Insights into potential risk factors for PKDL have improved in recent years and include the importance of the drug regimen used for treating the preceding VL episode and the skin pharmacokinetics of the drug. However, these risk factors may differ between EA and the SEAR [10–12]. In Sudan, the younger age of patients and the short interval between VL and PKDL (12 months) in these patients may be attributed to their developing immune response, as these children have lower frequencies of T helper (Th)1, Th2 and Th17 cells and a greater number of T-regulatory cells, which translates into the persistence of parasites in the skin during an episode of VL [13–16]. In SEAR, the long lag period and the generally older age of patients (adolescents/young adults) suggest that repeated exposure to *Leishmania* or intercurrent co-infections may play a major role and influence the immune response, but this needs to be established [8].

Other risk factors common for all endemic areas include environmental exposure (e.g., UV light, arsenic), parasite-related factors, such as co-infection with symbionts (e.g. *Leptomonas seymouri*) and vector-related factors (e.g., sand fly saliva, co-infections ( e.g. with *L. seymouri* narna-like virus 1 [Lepsey NLV1]). Additionally, ethnic differences and genetic factors (PKDL is associated with decreased function of the interferon-gamma receptor 1 gene [*IFNGR1*]) [17–21]. Taken together, these findings suggest that all risk factors are associated with modulation of immune responses following *Leishmania* infection.

PKDL is considered to play an important role in transmission, particularly during inter-epidemic periods. Recent studies from the SEAR and EA show that active VL, PKDL and VL–human immunodeficiency virus (HIV) cases contribute to sand fly-mediated parasite transmission. Nodular PKDL lesions have been demonstrated to be more infectious than macular lesions. In India, VL patients co-infected with HIV presented a threefold increased risk of infecting sand flies compared with non-co-infected patients, whereas both asymptomatic and VL patients who had been cured were not found to be transmitters. Molecular xenomonitoring and characterization of *Leishmania donovani* in India revealed a high infection rate in* Phlebotomus* sand flies (*Ph. argentipes* and *Ph. longiductus*) and the presence of *Leishmania* DNA in other sand fly species (including *Ph. papatasi* and *Ph. sergenti*). Definitive evidence for an animal reservoir is still lacking, although dogs in xenodiagnosis experiments were able to infect sand flies, based on PCR outcomes [22–24].

## Session 2: Immunology of PKDL (Presenters: Susanne Nylen, Ahmed Musa*, Ritika Sengupta, Madhurima Roy) *could not attend last minute; summary of presentation is included

In the SEAR, PKDL patients present in equal proportions with macular or polymorphic lesions. Similar to vitiligo, macular PKDL lesions are associated with the disruption of melanogenesis, with the hypopigmented border demonstrating infiltration of CD8^+^ T cells [25]. Various immune factors, such as increased levels of proinflammatory cytokines (e.g., interferon gamma [IFN-γ], interleukin 6 [IL-6], tumor necrosis factor alpha [TNF-α]), together with increased levels of T cells and neutrophil-associated chemokines (IL-8, IL-17, CXCL9/10), can damage melanocytes in a single manner or in a concerted manner, and a strong association with an increased presence of lesional CD8^+^ T cells has been demonstrated [26]. These CD8^+^ T cells are IL-10^+^, cytotoxic T-lymphocyte-associated protein 4 [CTLA4]^+^ and TNF-α, and demonstrate features of immune exhaustion. An enhanced expression of *Perforin-1*, *Granzyme B/H/K* and *granulysin* was observed in lesional borders of both forms of PKDL, nodular >>> macular, suggesting potential cytotoxic features that could mediate the destruction of lesional melanocytes. In terms of markers of the melanogenesis pathway, nodular PKDL lesion borders showed a reduced expression of the tyrosinase gene (*TYR*), similar to vitiligo, whereas macular PKDL lesion borders displayed no change. In PKDL lesions, melan-A levels were significantly diminished in both forms of lesions, macular >>>> nodular. Furthermore, in PKDL lesions, the expression of the TYR enzyme and the MITF transcription factor was downregulated at both the gene and protein levels, indicating a likely impairment of the melanogenesis pathway.

Other immune players included the presence of an activated inflammasome (presence of NLRP3 and caspase 1 together with with IL-1β) in epidermal keratinocytes, along with activated neutrophils (CD66b^+^/CD64^+^) at lesional sites that secrete tissue-damaging molecules (e.g. myeloperoxidase, neutrophil elastase, matrix metalloprotease 9). These players were proposed as possibly disrupting the extracellular matrix (e.g. collagen-1), thereby facilitating immune cell infiltration into the lesion sites [26].

In a study in Sudan, treatment with PKDL in combination with paromomycin + miltefosine (MF) resulted in a cure rate of 98% and induced a Th1/Th2/Th17 response, whereas treatment with liposomal amphotericin B (LAmb) + MF resulted in a cure rate of 80% along those with a Th1/Th2 response. In those who relapsed, the concentrations of IFN-γ, TNF and IL-1β at diagnosis were lower than respective concentrations non-relapsing patients, suggesting their potential to be used as immune markers for predicting outcomes. Treatment effectiveness was related to increased production of IFN-γ and the absence of IL-10 [27, 28].

In EA, CD4^+^ T cells play an important role in PKDL; during parasitic infection, CD4^+^ T cells are suppressed, and removal of T-regulatory cells does not improve CD4^+^ T-cell activation. IL-10 is an important suppressor of CD4^+^ T-cell function, especially in those patients who produce IFN-γ. CD4^+^ and CD8^+^ T cells play dual roles, contributing to infection control and immune dysfunction. The identification of markers of T-cell exhaustion (e.g. IL-10, protein receptor CTLA4 and inhibitory receptor LAG-3) identified in VL highlighted the complexity of immune regulation. In PKDL in the SEAR, distinct immune profiles were observed between macular and nodular lesions, with nodular PKDL exhibiting elevated IL-10 and TNF messenger RNA (mRNA) levels, indicating severe pathology. Comparative findings with those of autoimmune disorders such as vitiligo highlighted similarities in immune cell infiltration, suggesting avenues for therapeutic exploration in delineating the pathogenesis of infectious hypopigmentation.

Studies have demonstrated that the phenotypes of CD4^+^ T-cell subsets in peripheral blood do not differ between PKDL patients and endemic controls. LAG-3 (lymphocyte activation gene-3), an important checkpoint molecule, can be targeted for drug therapy. IFN-γ and IL-10 production significantly increased after simulation laboratory assessment (SLA) via a whole-blood assay (WBA) in PKDL patients, implying that WBA may be considered an immunological diagnostic tool for PKDL. Although PKDL manifests primarily through skin-resident parasites, thereby reflecting the role of local immunity in pathology, the *Leishmania*-specific response in WBA suggests that peripheral blood cells remain exposed to antigens and retain immunological memory [29].

## Session 3: vaccines and immunotherapy against PKDL (Presenters: Abhay Satoskar, Ahmed Musa*, Rajiv Kumar) * could not attend last minute, summary of presentation included

The current focus is on developing a live-attenuated *Leishmania* parasite vaccine aimed at preventing disease. The rationale behind vaccination is based on experience with leishmanization, where previous infection (i.e. exposure to all *Leishmania* antigens rather than a selection of peptides and a degree of parasite persistence) may maintain immunological memory to prevent new infection resulting in disease. The centrin gene-deficient *L. donovani* (LdCen−/−) vaccine is promising, as it induces similar immune responses. However, concerns of visceralization with a live attenuated *L. donovani* vaccine persist. A second-generation leishmanization dermatotropic *L. major* vaccine (LmCen−/−) was developed that showed good protection in animal models [30]. To further determine vaccine efficacy, a controlled human infection model (CHIM) using *L. major* may be used as an alternative to large-scale prospective phase III field studies, given the low number of VL cases in THE SEAR and the current political situation in Sudan.

Immunochemotherapy and immunotherapy for PKDL have been tested in Sudan, with variable responses. The first-generation vaccine (alum/ALM + BCG) provided in combination with SSG to patients with PKDL showed promising results in a proof-of-concept study, but vaccine production was stopped by the WHO as the crude vaccine was difficult to standardize [31]. A second-generation vaccine with SSG did not show any promise, but changing Th2 responses were observed (increased IL-5). A third-generation vectored vaccine alone (ChAd63-KH) showed promise in phase 2a but this potential was not substantiated by a phase 2b study [32, 33].

The best biomarker for vaccine efficacy is the measurement of cell-mediated immunity; from previous vaccine field-based studies, the leishmanin skin test (LST) seems to be an appropriate tool [34]. As the former *L. infantum* skin antigen test is no longer available, a new LST has been developed from *L. donovani*; this freeze-dried product, which adheres to good manufacturing practices (GMPs), is scalable and cost effective for assessing immunogenicity and could serve as a diagnostic biomarker for monitoring vaccine responses [35]. The *L. major* vaccine (LmCen−/−) and the LST antigen were developed in parallel, and preclinical development and good laboratory practice (GLP) toxicology studies have been completed.

CD4^+^ T cells play a crucial role in parasitic immunity, with gene signatures that predict disease outcomes in PKDL. Increased IFN-γ and IL-10 production upon SLA stimulation highlights the occurrence of systemic protective immune responses in PKDL patients. In VL, LAG-3 was identified as a potential host-directed therapy checkpoint target. PKDL is driven primarily by skin-resident parasites, but systemic immune responses are also evident, as patients with PKDL show antigen-specific memory responses. In this context, the NGFR and CCL3 markers distinguished polymorphic from nodular PKDL, potentially aiding in the development of personalized therapeutic strategies.

## Session 4: *Leishmania* co-infections (presenters: Shalindra Ranasinghe, Prasanta Saini, Sutopa Roy)

Genetic diversity in *Leishmania* strains has been demonstrated in VL and cutaneous leishmaniasis (CL) dynamics in Kerala, India and Sri Lanka; the *L. donovani* MON-37 strain causes both VL and CL, resulting in genetic divergence. Genetic hybridization between *L. donovani*, *L. major* and *L. tropica* was demonstrated via whole-genome sequencing (WGS). There is evidence of genetic hybridization between the viscerotropic and dermatotropic strains of *L. donovani* in the natural vector *Ph. argentipes*.

While PKDL in the SEAR seems restricted to VL caused by *L. donovani*, in Sri Lanka, a genetically different strain with single nucleotide polymorphisms (SNPs) (*mTOR*) and copy number variations (*A2* gene) causes CL. A similar diversity was reported from Kerala, India, where VL was reported from the central part of the state, whereas cases of CL were reported in two clusters among tribal populations residing in hilly forest areas, both caused by *L. donovani* MON-37, with *Ph. argentipes* as the vector and dogs as the potential reservoir [36]. However, no Leishman-Donovan (LD) bodies were identified in the splenic aspirates sourced from these dogs [36].

In a nation-wide molecular xenomonitoring of *L. donovani* from different biogeographical zones of India, six* Phlebotomus* species (*Ph. argentipes*, *Ph. papatasi*, *Ph. sergenti*, *Ph. longiductus*, *Ph. bruneyi*, *Ph. major)* were identified to be naturally infected with *L. donovani*, suggesting a wide distribution and circulation of *L. donovani* in India.

As HIV-VL co-infection exacerbates immune suppression, progression of HIV can lead to more severe forms of VL; conversely, VL may also accelerate HIV progression due to immune suppression. Challenges in co-infection include pauci-cellular bone marrow, rendering tissue aspirates less reliable and cross-reactivity in diagnostics and, therefore, misinterpretation of test results. Serological tests are less sensitive in co-infected patients, and the results of tests are equivocal due to factors such as test format, region of endemicity and level of immunosuppression.

The lesions of PKDL are often indistinguishable from other dermatoses like leprosy, and as both diseases can occur in overlapping endemic areas, clinical decision-making is often challenging. Traditional methods of detection often falter, especially when parasite and/or bacillary load is low, emphasizing the need for availability of sensitive and specific molecular tools. Six cases of PKDL and leprosy co-infections were presented for which molecular tools such as quantitative PCR (qPCR), semi-quantitative PCR and Sanger sequencing were employed to confirm this co-infection.

## Session 5: diagnosis of PKDL (presenters: V. Ramesh, Shalindra Ranasinghe, Ruchi Singh, Ashish Kumar, Ahmed Abd El-Wahed, Kristien Cloots, Jose-Antonio Ruiz-Postigo)

The diagnostic dilemma with PKDL is the challenge to distinguish it from other common NTD skin diseases, with leprosy being the most common [37]. Other important differential diagnoses include lichen scrofulosum (tuberculosis), pityriasis alba, pityriasis versicolor and vitiligo. Treatment guidelines for co-infected patients should be developed in coordination with the National Vector Borne Disease Control Programme, Govt. of India. Following the implementation of active surveillance, the macular form of PKDL was demonstrated to be as common as the polymorphic form (1:1), whereas in passive surveillance, i.e. hospital-based reports, the polymorphic form was more common (9:1 ratio). Clinically, the distribution may be localized, generalized, acral, centripetal or extensive. The characterization of macular PKDL is based on varying degrees of pigment loss, with a centrifugal distribution; in contrast, in vitiligo, the distribution of depigmentation is centripetal. Macules in PKDL may be confluent and may occur in combination with papules or nodules (polymorphic form). The histopathology and immune profiles of macular and polymorphic PKDL differ [38, 39]. Less well-known features of macular PKDL are that the lesions may be infectious to sand flies (<10%) and that the number of *Leishmania* parasites is not proportional to the extent of the lesion. The LST results remain negative for macular PKDL, in contrast to patients with treated VL where nearly all test results become positive. The detection of parasites in macular lesions by Giemsa staining has poor sensitivity; therefore, qPCR is a better tool and urgently needs validation.

A CORE outcome measure instrument has been developed for localized CL (“LeishCOM_LCL”) to standardize the assessment of CL wound healing, clinical cure, treatment efficacy and impact on quality of life via clinical trial data. This instrument tracks parameters such as palpability, ulcer area reduction and investigator-assessed scarring over time [40]. In the absence of quantifiable tools for monitoring PKDL, this approach could be considered.

The skin microbiopsy tool is minimally invasive, painless and leaves no scar; in an Ethiopian study, this device detected significantly more *Leishmania* DNA among asymptomatically infected individuals than did finger prick blood samples [41]. The sensitivity for PKDL lesions was 85% (94% for the nodular type and 70% for the macular type) [36]; for CL lesions, sensitivity was 69–100% [40–43]. Challenges in implementing this tool include the possibility that parasite distribution may be heterogeneous; in addition, the technique still requires PCR, and the cost is € 10–15 for the sampling device alone [41–44]. The CL Detect™ Rapid Test (InBios Int., Seattle, WA, USA) is an immunochromatographic assay that detects *Leishmania*-targeting peroxidoxin antigen in ulcerative skin lesions. The material for testing is collected by scraping the lesions, and the detection limit is 312–5000 parasites. The test was found to have a sensitivity of 73% in PKDL, compared with 76% for detecting LD bodies in histopathological samples and 89% for the rK39 rapid test [38].

Molecular assays require field applicability to increase access to these tools, and several options are now under study for molecular diagnosis in the field [44, 45]. Using qPCR and loop-mediated isothermal amplification (LAMP) assays in peripheral blood, the sensitivity for diagnosing PKDL (macular and polymorphic cases combined) was 69–80%, with qPCR performing slightly better than LAMP; the specificity for both molecular tests was 93–100% [46, 47]. The multiplex LAMP test for kinetoplast (kDNA; for VL) and 18S ribosomal DNA (rDNA; for CL) showed a sensitivity of 92–100% and a specificity of 99–100% for VL and 86% for CL. Another field-friendly application of molecular diagnostics is the mobile suitcase laboratory, which uses the *L. donovani* recombinase polymerase assay (RPA) and allows the readout result to be sent to a smartphone. Nucleic acid extraction via SwiftX takes 15 min, and nucleic acid identification also takes 15 min; all reagents are stable below 40 °C for 3 months. A pan-*Leishmania* PCR was developed and tested against qPCR; the sensitivity was 91% and the specificity was 96%. In PKDL, good concordance was found with qPCR as a test of cure; similarly, qRPA demonstrated good concordance with qPCR. RPA can be used to detect LD bodies in samples obtained by xenodiagnosis, but it is field applicable and less expensive than qPCR is (USD 8–9 vs. 30, respectively) [48]. A portable smartphone-based molecular test ‘Minoo’ has been developed; this test was reported to have an overall clinical sensitivity of 88% and specificity of 91% in 174 samples tested from India and Bangladesh [49]. Oxford nanopore sequencing and Sanger sequencing were compared for the differentiation of closely related *Leishmania* species. Oxford Nanopore is also being assessed for its suitability as field-adaptable technology.

Studies with PKDL isolates demonstrated a significant increase in drug susceptibility toward SAG after the introduction of MF. In contrast, there was no difference in susceptibility to MF among the PKDL isolates before and after the introduction of MF, suggesting no change in the natural susceptibility of *L. donovani* to MF. However, isolates from patients who relapsed showed a significant decrease in susceptibility to MF, suggesting the emergence of MF-resistant parasites. The susceptibility of PKDL isolates to AmBisome (AmB) remained unchanged after the introduction of MF, but the half maximal inhibitory concentration (IC_50_) values were higher in the post-MF era [50].

The WHO’s digital platform is aimed at aiding in the differential diagnosis of skin conditions, analyzing lesion images at high speed and providing offline accessibility. Artificial intelligence (AI) systems integrate databases and algorithms to provide tailored diagnostic support for NTDs. These AI-based diagnostic tools face limitations due to insufficient image datasets (e.g. for macular PKDL), suggesting that more data are needed to increase algorithm accuracy and training. There are as yet no data on the development of AI to evaluate PKDL after treatment, and this is an urgent need, especially for those who are not experts in dermatology.

## Session 6: treatment of PKDL (presenters: Shyam Sundar, Krishna Pandey, Fabiana Alves, Thomas Dorlo, Eduard E. Zijlstra)

The current treatment for PKDL in the SEAR is 12 weeks of MF; this long treatment duration causes frequent toxicities in patients (including ocular ones) that may result in incomplete treatment and loss to follow-up. Studies from India reported that treatment with MF 2.5 mg/kg or 100 mg/day for 12 weeks was superior to treatment with LAmB at 30 mg/kg total dose (administered in 6 doses of 5 mg/kg, biweekly for 3 weeks). The follow-up period was 12 months, and a clinical assessment was performed [51].

A non-comparative, randomized trial from India and Bangladesh in PKDL reported that the efficacy of LAmB (total dose of 20 mg/kg, administered at doses of 4 mg/kg on days 1, 4, 8, 11 and 15) was 90% at 12 months and 83% at 24 months [52]. This result was comparable to the efficacy of LAmB (total dose of 20 mg/kg, administered at doses of 4 mg/kg on days 1, 4, 8, 11 and 15) in combination with MF (oral route [PO], twice daily [b.i.d.] for 21 days, allometric dosing), with 85% efficacy at 12 months and 88% efficacy at 24 months. Compared with the combination therapy, LAmB monotherapy resulted in more relapses (1.7% vs 8.0%, respectively). The assessment was qualitative when the results were plotted in boxes on a manikin. From a safety perspective, the frequency of adverse events (AEs) in the LAmB + MF combination arm was greater (78%), mostly due to gastrointestinal AEs.

A noncomparative randomized trial from Sudan in PKDL of ≥ 6 months duration or grade 3 PKDL reported that the cure rate with paromomycin (PM; 20 mg/kg/day, intramuscular injection [IM] for 14 days) + MF (PO, b.i.d. for 42 days, allometric dosing) combination therapy was 100% at 12 months, whereas the efficacy of LAmB (20 mg/kg total dose, 5 mg/kg intravenous [IV] on days 1, 3, 5 and 7) + MF (PO, b.i.d. for 28 days, allometric dosing) combination therapy was 86% at 12 months. The assessment was qualitative when the results were plotted in boxes on a manikin. There were no serious AEs (SAEs) reported, but the proportion of AEs in the LAmB + MF combination arm was greater than that in the PM + MF combination arm (51% vs 24%, respectively) [28].

New compounds being studied include LXE408, which is currently in phase II trials in India and Ethiopia. Testing of CpG-D35, an immunomodulatory agent, was stopped in phase 1b trials in Colombia because of feasibility; strategic development is under review. Monitoring the drug concentrations of LAmB and MF in plasma and skin may be an efficient tool for guiding new treatment regimens: while MF exhibited higher skin-to-plasma ratios, LAmB demonstrated prolonged skin retention, thus supporting tailored regimens based on drug pharmacokinetics.

Biomarker-based approaches for determining treatment outcomes may include clinical (development of a disease activity score, if possible), serological, immunological (Th1/Th2 cytokine ratio) and parasitological parameters (microscopy, qPCR). IFN-γ and IL-10 can be measured in the blood and reflect dermal immune responses. The challenge is to define the optimal time point to assess the success of treatment via a combination of clinical, immunological and/or parasitological approaches. Of these, an immunological biomarker that can be measured in the blood is preferred as the optimal marker for a cure, whereas clinical assessment may lead to overtreatment (particularly in macular PKDL, where parasite elimination and repigmentation do not occur simultaneously), whereas a parasitological marker requires repeated sampling of the skin, which is logistically challenging. Three-dimensional optical scanning may provide an accurate, field- and user-friendly tool as it measures the diameter, circumference, volume and color change of each individual lesion; calculates the difference from baseline or preceding scans of an essentially unlimited number of lesions [53]; and, importantly, can be performed with a smartphone.

## Recommendations/key take-home messages (presenters: Ritika Sengupta, Kristien Cloots)

### Epidemiology and control

- Continued active surveillance for VL and PKDL patients.
- To address and curtail risk factors, the role of the development of PKDL in the SEAR should be explored in depth, considering clinical, parasitological, immunological, ethnic, genetic and environmental factors.
- Transmission, clarifying the role of an animal reservoir, if any, in the SEAR and EA and describing the relative contribution to transmission compared with VL, VL-HIV and PKDL cases.

### Immune responses, vaccines and immunotherapy

- We describe and compare the variable immune responses between PKDL in the SEAR and EA, as well as the different forms, with the aim of understanding their pathophysiology and suggesting suitable intervention(s).
- Research into systemic and localized immune responses in PKDL is needed to refine immunotherapeutic approaches.
- Anti-inflammatory and immune-restorative therapies for managing hypopigmentation and reducing lesion severity should be explored. Therapies based on models of vitiligo should be investigated to improve outcomes in PKDL-associated pathology.
- Exploration of the LmCen−/− vaccine for use in addition to VL/PKDL drug therapy to prevent and/or treat PKDL.

### Parasite diversity

- Genetic studies are also needed to understand strain-specific virulence and adapt control strategies.
- The role of *Leishmania* parasites infected with symbionts such as *Leptomonas seymouri* should be clarified.

### Diagnosis and biomarkers

- Standardize a universal qPCR protocol and invest in cost-effective, scalable diagnostic platforms for broader applicability.
- Encourage the availability of field-applicable molecular diagnostic technologies such as RPA and minimally invasive diagnostics with microbiopsies.
- Expanding data collection efforts for AI-based diagnostic tools should focus on underrepresented challenging conditions such as macular PKDL. The aim of this study is to validate AI algorithms for differential diagnosis in resource-limited settings.
- The LeishCOM_LCL tool was compared with other diagnostic tools in endemic regions.
- The complete development of LST as a marker for cell-mediated immunity for use in phase III vaccine studies will lead to the validation and standardization of advanced biomarkers, such as INF-γ/IL-10 in peripheral blood, via qPCR and cytokine profiling.

### Treatment of PKDL

- Implement new guidelines for VL and PKDL in the SEAR and EA (once published); for VL, explore combinatorial regimens with a short duration leveraging pharmacokinetic insights for safety and improved efficacy, such as decreasing the transition from VL to PKDL and minimizing relapse; for PKDL, aim to replace parenteral drugs with oral drugs for a more patient-friendly, ambulatory treatment option.
- Expand/explore the therapeutic arsenal for VL and PKDL from parasite-killing drugs (including all drugs currently in use) and/or immune modulators (including CpG-D35 and vaccines, still under investigation), as well as immune checkpoint inhibitors (e.g. LAG-3, expressed by exhausted T cells) [54].

## Supplementary Information

**Additional file 1: Text S1.** Agenda Seventh Post Kala-Azar Dermal Leishmaniasis (PKDL) Consortium meeting.

**Additional file 2: Text S2.** List of presenters at Post Kala-Azar Dermal Leishmaniasis (PKDL) Consortium meeting.

**Additional file 3: Text S3.** Regional priorities, regional pillars of progress and challenges for disease elimination.

## Data Availability

Data supporting the main conclusions of this study are included in the manuscript.

## Abbreviations

AI: Artificial intelligence
AmB: AmBisome
CL: Cutaneous leishmaniasis
DND*i*: Drugs for Neglected Diseases* initiative*
EA: East Africa
GLP: Good laboratory practice
GMP: Good manufacturing practice
IFNGR1: Interferon-gamma receptor 1
LAG-3: Lymphocyte activation gene-3
LAmB: Liposomal amphotericin B
LAMP: Loop-mediated isothermal amplification
LST: Leishmanin skin test
MF: Miltefosine
NTD: Neglected tropical disease
PKDL: Post-kala-azar dermal leishmaniasis
RPA: Recombinase polymerase assay
SEAR: Southeast Asia region
SSG: Sodium stibogluconate
VL: Visceral leishmaniasis
WBA: Whole blood assay
