# Publisher Correction: Report of the Seventh Post-Kala-Azar Dermal Leishmaniasis Consortium Meeting, Kolkata, India, 28–29 November 2024

**DOI:** 10.1186/s13071-026-07422-0

**Published:** 2026-05-28

**Authors:** Mitali Chatterjee, Ritika Sengupta, Kristien Cloots, Eduard E. Zijlstra

**Affiliations:** 1 Department of Pharmacology, Institute of Postgraduate Medical Education and Research (IPGMER), Kolkata, India; 2 Department of Public Health, Institute of Tropical Medicine, Antwerp, Belgium; 3 Rotterdam Center for Tropical Medicine, Bovenstraat 21, 3077 BB Rotterdam, The Netherlands

**Publisher Correction: Parasites & Vectors (2026) 19:115** 10.1186/s13071-025-07175-2

Following publication of the original article, it has been brought to the journal's attention that miscellaneous correction requests were not implemented during production of the article. The corrections have now been implemented (please see the annotations in Additional file 1 for the details of these corrections). The publisher thanks you for reading this erratum and apologizes for any inconvenience caused.

## Supplementary Information

Below is the link to the electronic supplementary material.

Additional file 1.

