# Supplementary material for: Report of the Seventh Post-Kala-Azar Dermal Leishmaniasis Consortium meeting, Kolkata, India, 28–29 November 2024
Source: Parasit Vectors. 2026 Mar 17;19:115. doi: 10.1186/s13071-025-07175-2 (PMC12994256; doi:10.1186/s13071-025-07175-2)
Supplement: Supplementary file 1 — Additional file 1: Text S1. Agenda Seventh Post Kala-Azar Dermal Leishmaniasis (PKDL) Consortium meeting. [file 13071_2025_7175_MOESM1_ESM.doc]

# 7^th^ Post Kala Azar Dermal Leishmaniasis (PKDL) Consortium Meeting

# November 28-29, Kolkata, India

Day-1 Agenda - 28^th^ November 2024

| **8:00-8:30** | **Registration** | **All** | **30 min** |
| --- | --- | --- | --- |
| **8:30-8.45** | **Opening Remarks** |  |  |
|  | Welcome & Objectives of the meeting | **Mitali Chatterjee**  *(IPGME&R, Kolkata, India)*  **Ed E Zijlstra**  (*Rotterdam Centre for Tropical Medicine, Netherlands*) | 15 min |
| **8.45-10.30** | **Epidemiology & control -1** | **Chair: Shyam Sundar, Ed Zijlstra** | **1 h 45 min** |
|  | Strategic shift to achieve SDG target 3.3 towards elimination of communicable diseases in South East Asia region-WHOs vision and priorities | **Suman Rijal**  *(WHO SEARO)* | 20 min |
|  | Global burden of PKDL | **Saurabh Jain**  *(WHO, Geneva) Pre-recorded* | 10 min |
|  | Status of VL control in Indian subcontinent ISC –  focus on PKDL | **Aya Yajima**  *(WHO SEARO)* | 15 min |
|  | PKDL burden - Bangladesh | **Dinesh Mondal,**  *(icddr’b*, *Bangladesh)* *Pre-recorded* | 10 min |
|  | Status of VL control in Africa – focus on PKDL | **Abate Beshah**  *(WHO, Africa)* *Pre-recorded* | 15 min |
|  | The burden of PKDL in Sudan | **Ed E Zijlstra, Brima Musa Younis***  *(Rotterdam Centre for Tropical Medicine, Netherlands*  **Institute of Endemic Diseases, Univ. of Khartoum, Sudan)* | 15 min |
|  | Discussions | All | 20 min |
| **10:30-11:00** | **Tea/Coffee Break** |  | **30 min** |
| **11:00-12:50** | **Epidemiology & control 2** | **Chair: Suman Rijal, SK Guha** | **1h 50 min** |
|  | Risk factors for PKDL | **Ed Zijlstra**  *(Rotterdam Centre for Tropical Medicine, Netherlands)* | 20 min |
|  |  | **Jose Postigo**  *(WHO, Geneva) Pre-recorded* | 20 min |
|  | Update on entomological  studies and zoonotic transmission in Eastern Africa | **Dia Elnaiem**  *(University of Maryland, USA) Pre-recorded* | 20 min |
|  | Xenodiagnosis; progress and future prospects | **OP Singh**  *(Banaras Hindu University, Varanasi, India)* | 20 min |
|  | Discussions | All | 30 min |
| **12:50-14:00** | **Lunch** |  | **1 hr** |
| **14:00-15:30** | **Immunology** | **Chair: Abhay Satoskar, Syamal Roy** | **1 hr 30 min** |
|  | Update on pathogenesis in VL and PKDL | **Susanne Nylen**  *(Karolinska Institutet,Stockholm, Sweden)* | 20 min |
|  | Cellular immune responses during and after treatment for PKDL in Sudan | **Ahmed Musa**  *(University of York, UK) Pre-recorded* | 20 min |
|  | Unraveling the ‘pale puzzle’: an attempt to decode the pathogenesis of hypopigmentation in PKDL | **Ritika Sengupta**  **Madhurima Roy**  *(IPGME&R, Kolkata, India)* | 10 min  10 min |
|  | Discussions | All | 30 min |
| **15:30-16.00** | **Tea/Coffee Break** |  | **30 min** |
| **16.00-17:30** | **Vaccines and immunotherapy** | **Chair:** **Chitra Mandal,** **A Selvapandiyan** | **1 hr 30 min** |
|  | Update on the use of vaccines for the prevention and treatment of leishmaniasis with focus on VL and PKDL | **Abhay Satoskar**  *(Ohio State University, USA)* | 20 min |
|  | Lessons learnt from vaccine studies in Sudan | **Ahmed Musa**  *(University of York, UK) Pre-recorded* | 20 min |
|  | Immunological signature of CD4^+^ T cells in PKDL | **Rajiv Kumar**  *(Banaras Hindu University,Varanasi, India)* | 20 min |
|  | Discussions | All | 30 min |
| **17:30** | **Inauguration** |  |  |
| **19:00-22:00** | **Dinner** | **Cafe Ekante, Houseboat restaurant** |  |

**Day-2 Agenda – 29^th^ November 2024**

| **09:00-10:30** | ***Leishmania* and co-infections** | **Chair:** **Jaya Chakravarty***,* **Yogiraj Roy** | **1 hr 20 min** |
| --- | --- | --- | --- |
|  | Genetically different *Leishmania donovani* induced Cutaneous Leishmaniasis in Sri Lanka and a new tool (LeishCOM_LCL) to assess healing | **Shalindra Ranasinghe**  *(University of Sri Jayewardenepura, Sri Lanka)* | 15 min |
|  | Genetic diversity of *Leishmania donovani* causing dual clinical manifestations (VL/CL) in Kerala, India | **Prasanta Saini**  *(ICMR-Vector Control Research Centre, Puducherry, India)* | 15 min |
|  | HIV/VL Co-infections and challenges in treatment | **Krishna Pandey**  *(ICMR-RMRIMS, Patna, India)* | 10 min |
|  | Can two skin NTD's co-exist? Spotlight on PKDL and Leprosy | **Sutopa Roy**  *(IPGME&R, Kolkata, India)* | 10 min |
|  | Discussions | All | 30 min |
| **10:30-11:00** | **Tea/Coffee Break** |  | **30 min** |
| **11:00-13:00** | **Diagnosis** | **Chair:** **Pradeep K Das, Fabiana Alves** | **2 hrs** |
|  | Clinical Features and Differential Diagnosis of macular PKDL | **V Ramesh**  *(Safdarjang Hospital & VMMC,New Delhi, India)* | 15 min |
|  | Molecular tools for diagnosis of PKDL | **Ruchi Singh**  *(ICMR-National Institute of Pathology,*  *New Delhi, India)* | 15 min |
|  | qPCR for monitoring PKDL | **Ashish Kumar**  *(ICMR-RMRIMS, Patna, India)* | 10 min |
|  | Field applicable tools for diagnosis and monitoring PKDL | **Ahmed Abd El Wahed**  *(University of Leipzig, Germany)* | 15 min |
|  | Use of microbiopsies in Leishmaniasis | **Kristien Cloots**  *(Institute of Tropical Medicine, Antwerp, Belgium)* | 10 min |
|  | AI in diagnosis and image repository of Skin NTDs | **Jose Postigo**  *(WHO, Geneva)Pre- recorded* | 15 min |
|  | Discussions | All | 30 min |
| **13:00-14:00** | **Lunch** |  | **1 hr** |
| **14:00-15.45** | **Treatment** | **Chair:** **Shyam Sundar, Bibhuti Saha** | **1 hr 45 min** |
|  | Recent recommendations for treatment of PKDL in Indian subcontinent and Eastern Africa | **Shyam Sundar**  *(Banaras Hindu University, Varanasi, India)* | 20 min |
|  | Efficacy and safety of liposomal amphotericin B (AmBisome) versus miltefosine in patients with post-kala-azar dermal leishmaniasis. | **Krishna Pandey**  *(ICMR-RMRIMS, Patna, India)* | 10 min |
|  | Update: new chemical entities (LXE, DNDi pipeline) | **Fabiana Alves**  *[Drugs for Neglected Diseases Initiative (DNDi), Geneva, Switzerland]* | 20 min |
|  | Optimizing treatment for PKDL: novel insights from skin target site PK studies and modelling of drug exposure-parasite-lesion relationships | **Thomas Dorlo**  *(Uppsala University, Sweden)* | 20 min |
|  | Challenges in monitoring treatment in PKDL; need for innovation | **Ed Zijlstra**  *(Rotterdam Centre for Tropical Medicine, Netherlands)* | 15 min |
|  | Discussions | All | 20 min |
| **15.45-16.00** | **Tea** |  | **15 min** |
| **16:00- 17:30** | **Final discussion and Conclusions** | **Chair: Scientific Advisory Committee** | **1 hr 30 min** |
|  | Rapporteurs report | Rapporteurs | 2 x15 min |
|  | Discussion | All | 30 min |
|  | Recommendations | All | 30 min |
| **17:30-18:00** | **Closing session** |  | **30 min** |
|  | Way forward for the PKDL Consortium |  | 15 min |
|  | Vote of thanks & Closing Remarks | **Mitali Chatterjee, IPGME&R, Kolkata** | 15 min |
