# Supplementary material for: Report of the Seventh Post-Kala-Azar Dermal Leishmaniasis Consortium meeting, Kolkata, India, 28–29 November 2024
Source: Parasit Vectors. 2026 Mar 17;19:115. doi: 10.1186/s13071-025-07175-2 (PMC12994256; doi:10.1186/s13071-025-07175-2)
Supplement: Supplementary file 3 — Additional file 3: Text S3. Regional priorities, regional pillars of progress and challenges for disease elimination. [file 13071_2025_7175_MOESM3_ESM.docx]

**Supplementary file 3 (from presentation by Yajima A)**

Regional priorities

- Strengthening and sustaining active case detection of KA and PKDL in low transmission settings
- Integration with other community outreach activities
- Integrated skin NTD screening/camps
- Integrated vector management with other VBDs
- Technological innovation: Case forecasting, geospacial mapping, xenomonitoring
- Ensuring universal access to diagnostics
- Special focus on high-risk areas: high number of child cases and emerging areas in Nepal
- Sporadic cases outside the endemic districts and endemicity assessment
- Cross-border notification and follow-up
- Sustaining entomological capacity in collaboration with malaria programme
- Addressing social and environmental determinants of health for sustained KA elimination (community hygiene, housing, health literacy

Regional Progress is based on:

- Pillar 1: Strengthening country ownership
  - Regional Partners Forum to advocate for sustained high-level commitments on NTDs
  - Launch of the Regional Strategic Framework on NTDs 2024-2030, aligned with the Global Roadmap
- Pillar 2: Accelerating programmatic actions
  - New RTAG for Kala-Azar and Malaria Elimination established
- Pillar 3: Intensifying integrated approaches
  - Convened Regional COR-NTD Meeting for South-East Asia
  - Supported integrated programme review of multi-diseases

(vector-borne diseases, skin NTDs) to facilitate identification of integration opportunities

- - Integrated Skin NTD Toolkit in SEAR

Challenges for Disease elimination include:

- Health system gaps
- Sustainable financing
- Health workforce
  - Case management capacity
  - Lab and diagnostic capacity (inc sero/genotyping)
  - Active case detection, contact tracing
  - Vector surveillance and control
- Health information system treatment and for vector control
  - Real-time surveillance system
  - Dashboard, case-based data, GIS maps
- Medical products and technologies
  - Universal access to drugs and RDTs
  - Vaccines feasible for public health use
  - Appropriate tools for diagnosis and treatment and for vector control

**Social and environmental determinants of health**

- Basic infrastructure
  - Poor housing and community sanitation
  - Roads, internet etc.
- Health literacy
  - Health awareness and seeking behaviours
- Equity
  - Gender, ethnic and social exclusion
- Climate change
  - Expanding endemicity to new areas
